# Hybrid Fluoro-Based Polymers/Graphite Foil for H_2_/Natural Gas Separation

**DOI:** 10.3390/ma16052105

**Published:** 2023-03-05

**Authors:** Angela Malara, Lucio Bonaccorsi, Antonio Fotia, Pier Luigi Antonucci, Patrizia Frontera

**Affiliations:** 1 Department of Civil, Energy, Environmental and Material Engineering, Mediterranea University of Reggio Calabria, 89124 Reggio Calabria, Italy; 2 National Interuniversity Consortium of Materials Science and Technology (INSTM), 50121 Florence, Italy; 3 CNR, Institute of Advanced Technologies for Energy “Nicola Giordano”—ITAE, 98122 Messina, Italy

**Keywords:** graphite, hydrogen separation, mixed gas, PVDF-HFP, membranes, Nafion^TM^

## Abstract

Membrane technologies and materials development appear crucial for the hydrogen/natural gas separation in the impending transition to the hydrogen economy. Transporting hydrogen through the existing natural gas network could result less expensive than a brand-new pipe system. Currently, many studies are focused on the development of novel structured materials for gas separation applications, including the combination of various kind of additives in polymeric matrix. Numerous gas pairs have been investigated and the gas transport mechanism in those membranes has been elucidated. However, the selective separation of high purity hydrogen from hydrogen/methane mixtures is still a big challenge and nowadays needs a great improvement to promote the transition towards more sustainable energy source. In this context, because of their remarkable properties, fluoro-based polymers, such as PVDF-HFP and Nafion^TM^, are among the most popular membrane materials, even if a further optimization is needed. In this study, hybrid polymer-based membranes were deposited as thin films on large graphite surfaces. Different weight ratios of PVDF-HFP and Nafion^TM^ polymers supported over 200 μm thick graphite foils were tested toward hydrogen/methane gas mixture separation. Small punch tests were carried out to study the membrane mechanical behaviour, reproducing the testing conditions. Finally, the permeability and the gas separation activity of hydrogen/methane over membranes were investigated at room temperature (25 °C) and near atmospheric pressure (using a pressure difference of 1.5 bar). The best performance of the developed membranes was registered when the 4:1 polymer PVDF-HFP/Nafion^TM^ weight ratio was used. In particular, starting from the 1:1 hydrogen/methane gas mixture, a 32.6% (v%) H_2_ enrichment was measured. Furthermore, there was a good agreement between the experimental and theoretical selectivity values.

## 1. Introduction

One of the main issues related to the widespread use and implementation of hydrogen (H_2_) as a usable energy vector is its distribution. Pipelines offer a cost-effective and effective way to move gaseous hydrogen with increased capacity and minimal energy loss when compared to other modes of transportation such as tube trailers and truck tankers [1]. In the last years, it has been argued as a possible key solution the transport in the already existing grids for the natural gas (NG); but then, its separation from NG remains a crucial question, especially when H_2_ concentrations higher than 20 v/v% are considered. Currently, natural gas is the primary energy carrier, and the transport capacity of the gas system is ten times larger than that of the electricity grid, inferring that the transport in the form of chemical fuel over long distances is more efficient than electricity [2,3,4,5].

The (re)-use of the existing NG network could serve as a “catalyst” for entering in the forthcoming hydrogen economy. Reusing natural gas pipelines for hydrogen transport presents four technical challenges. These include pipe material compatibility with high-pressure hydrogen gas environments, hydrogen/natural gas blending processing and pipeline operation, leakage and integrity management, safety, and end-user impact, despite the recent studies [6]. Quantifying the modifications would be necessary to transport hydrogen, e.g., just 5–10% of the costs inherent to a new pipeline; therefore, overall, energy transport through converted NG pipelines would result 100–200 times cheaper than a new electricity system. In comparison to newly dedicated hydrogen pipelines, hydrogen blending in the existing pipelines can save initial capital costs and lower delivery and maintenance costs [7]. Today, it is advised to maintain a hydrogen to natural gas blending ratio of less than 20% to guarantee pipeline safety and system stability as well as lower public risk [8]. However, considering the pipeline performance conditions and the practical hydrogen demands, the blending ratio can be increased [9]. H_2_ blending’s technical feasibility, effects, and mixing behaviour have been greatly studied [10,11,12,13]. Three gas-separation technologies could be used to extract hydrogen from mixtures in natural gas pipelines: pressure swing adsorption (PSA), membrane separation, and electrochemical hydrogen separation (EHS, or hydrogen pumping) [14]. In addition, a hybrid approach using a polymeric membrane and PSA was also studied to separate hydrogen (99.97%) from the (NG+H_2_) mixture with hydrogen contents lower than 10% [15].

In this context, crucial appears the role of innovative and performing materials [16] as well as of membrane technologies for hydrogen/methane separation, highlighting their potential role in the P2H roadmap [17]. The state of the art related to membranes for H_2_/NG separation includes a very large number of different materials. Briefly, the most performing ones are mainly based on dense metallic or ceramic structures, such as palladium alloy and proton conducting ceramics, respectively, able to separate pure hydrogen with impressive selectivity (>1000) but operating under severe working conditions, such as high temperature ranges of 300–900 °C, finally resulting prohibitive on account of their cost and energetic waste [18,19]. To date, polymeric membranes are considered the most promising technology for the reduced costs and the operating conditions compared to other solutions. The development of polymeric materials able to achieve the important combination of high selectivity, high permeability, mechanical stability, and processability at temperatures above 35 °C and pressures above several bars is a topic Issue for the distribution of hydrogen. Affordable resulted membranes are based on polymer of intrinsic microporosity (PIMs), metal organic frameworks (MOFs), mixed matrix membranes (MMMs), microporous inorganic membranes, zeolite imidazolate frameworks (ZIFs) and Troger bases [20,21,22,23]. Very different separation mechanism has been reported for each system, mainly based on solution–diffusion, molecular sieving and surface diffusion. In the mildest operating conditions, 35–50 °C and a pressure difference of 2–6 bar, the high selectivity exhibited by this material (66.5–142.7) was mainly ascribed to the favourable transport of small molecules, such as hydrogen, with increasing temperature [22,24,25,26].

Graphite-based materials have been demonstrated to exhibit a wide range of gas diffusion mechanisms dependent on pore structure parameters, such as pore diameter, open/closed porosity relation, and anisotropy of the structure. One of these materials, foil based on exfoliated graphite, is produced on an industrial scale as a sealing material and might potentially be used as microporous inorganic membrane for gas separation [17,22,23]

In this view, it is proposed to develop a new hybrid membrane supported over graphite foils (GF) and composed of fluoro-based polymers, such as Poly(vinylidene fluoride-co-hexafluoropropylene) PVDF-HFP and Nafion^TM^ [27,28,29]. PVDF-HFP is one among the most popular membrane materials due to its outstanding properties, including thermal stability, chemical resistance and excellent mechanical strength [30]. The key to the success of the separation process is the fabrication of suitable membranes yielding both high permeability and selectivity. Since the majority of polymer membranes suffer from the inherent disadvantage of the trade-off effect between permeability and selectivity, numerous physical and chemical methods have been developed to modify the existing polymer material in an effort to overcome the trade-off barrier and meet the practical application requirements. Blending and filling are two of the most often used techniques due to their simplicity and adaptability. Blending one polymer with another can drastically affect polymer chain mobility via intermolecular interaction, and subsequently can modify the permeation flux and the separation factor of blended membranes [17,31,32].

In this work, a preliminary evaluation of mechanical properties and separation performance of gases (pure methane without minor compounds and hydrogen) of hybrid polymer/graphite-based membranes is assessed at room temperature (25 °C) and near atmospheric pressure (using a pressure difference of 1.5 bar), taking also into account the influence of material preparation procedures.

## 2. Materials and Methods

**Figure 1:**
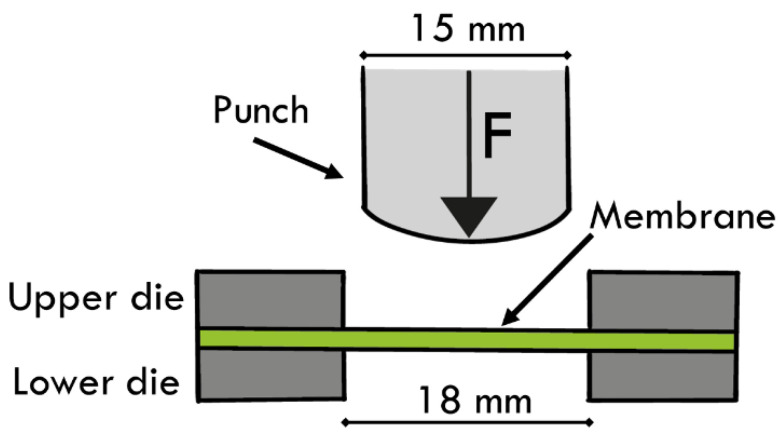
Small punch test configuration.

Commercial flat graphite foils (ProGraphite GmbH, Untergriesbach, Germany) of three different thicknesses, namely 1000 μm, 500 μm and 200 μm, were tested as carbon-based materials for the H_2_/CH_4_ gas separation. Organic polymers, such as fluoro-based co-polymer poly (vinylidene fluoride) PVDF-HFP (Sigma Aldrich, St. Louis, MO, USA) and Nafion (5wt.% in lower aliphatic alcohols/H_2_O, 15–20% water, Sigma Aldrich, St. Louis, MO, USA) as matrix and additive were used, respectively, to modify the pure graphite foils. Hybrid polymer-based membranes were realized using doctor blade technique in order to produce thin films on large surface areas. In essence, polymers were dissolved in acetone (ACS reagent, ≥99.5%, Sigma Aldrich, St. Louis, MO, USA), stirred for 8 h at 500 rpm and finally placed on the graphite substrate. A constant relative movement was established between the blade and the substrate, and the solution spread on the graphite foil to form a thin sheet which resulted in a gel-layer upon drying. Thin films were dried at room temperature for 24 h and finally treated for 1 h at 80 °C under vacuum. Prepared samples, summarized in Table 1, are labelled as the following: *G* for graphite support, *H*, *M*, *L* referred to the graphite thickness for, respectively, high (1000 μm), medium (500 μm) and low (200 μm), and *xx/yy* referred to polymer weight ratio. Obtained hybrid substrates were characterized in depth by complementary investigation techniques. Scanning electron microscopy (Phenom ProX, Deben, Suffolk, UK) was used to study membranes morphology and microstructure. Small punch tests (SPTs) were carried out to study membrane mechanical behaviour, reproducing the gas separation testing conditions. SPTs were conducted on circular-section samples with a diameter of 40 mm, using a Zwicki 2.5 kN machine coupled with a load cell of 200 N (Zwick/Roell, Ulm, Germany). The experimental setup consisting of a punch diameter of 15 mm, a hole in the lower die with a diameter of 18 mm and a displacement rate of 1 mm/min is depicted in Figure 1.

**Figure 2:**
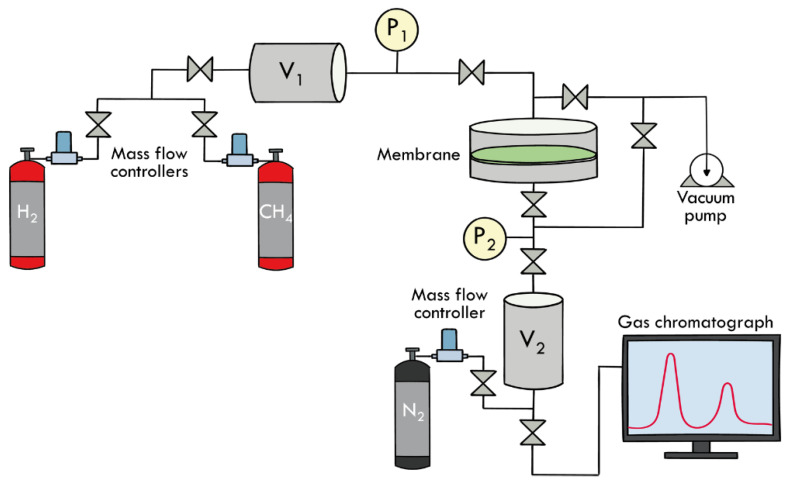
Schematic representation of the gas separation plant.

The gas separation activity of hydrogen/methane mixtures over membranes was investigated at ambient temperature and near atmospheric pressure conditions [33,34]. Membrane permeability was tested in an on-purpose permeation system as depicted in Figure 2, consisting of the gas mixture feeding section, the upstream gas circuit, the permeation cell, and the downstream section including the gas chromatograph.

The membrane permeability is measured according to the constant-volume, variable-pressure system, as described in the following. A gas mixture of CH_4_ and H_2_ at a fixed volume ratio is produced mixing the pure gases by calibrated electronic mass flow controllers (Brooks Instruments, Hatfield, PA, USA) and continuous feed to the permeation cell at the upstream pressure P_1_ = 1.5 bar, previously blended in the volume V_1_. The downstream volume V_2_ acts as permeate reservoir and is evacuated to vacuum (P_2_ = 10^−3^ bar) before each test. The pressure rise in the reservoir (P_2_) is recorded as a function of time and the test is stopped when the downstream pressure reaches 0.1 bar. The permeation cell is, then, separated by a valve and the reservoir pressure increased by adding a carrier gas (N_2_) up to 1 bar. The membrane samples were carefully evacuated using the high vacuum system before each test. The permeability P_A_ (Barrer) of a polymeric membrane of a given gas A is independent of the thickness of the membrane but is a property of the polymeric material and can be described by the product of a thermodynamic factor, the solubility coefficient S_A_ (m^3^/m^3^bar), and a kinetic parameter, the diffusion coefficient D_A_ (m^2^/s) calculated as reported by Koros et al. [35,36] and reported in Equation (1).

P_A_ = S_A_∙D_A_. (1)

In particular, the permeance (PM) is first defined as the gas volume which penetrates a certain membrane area per unit time at a given pressure difference and can be calculated from the permeate pressure increase according to Equation (2) where the value 3600 is the conversion factor for time [s/h], V_P_ is the permeate volume (m^3^), V_m_ is the molar volume of a gas at standard temperature and pressure (22.41 × 10^−3^ m^3^_STP_/mol at 0 °C and 1 atm), R is the universal gas constant (8.314 × 10^−5^ m^3^ bar/mol K), T is the absolute temperature (K), A is the exposed membrane area (m^2^), P_F_ (bar) is the feed pressure and P_P_ (bar) is the permeate pressures. Then, the permeability is defined as the product of the permeance and the membrane thickness (l).

(2) $$\mathrm{PM}=\frac{3600{\;V}_{P}V_{M}}{{\mathrm{RTAP}}_{F}}\frac{{\mathrm{dP}}_{P}}{\mathrm{dt}}.$$

The diffusion coefficient D can then be obtained by the “time lag” method, based on the penetration theory [34,36], Equation (3),

(3) $$\theta =\frac{l^{2}}{6D},$$

and subsequently the solubility is obtained from the steady state permeation and Equation (1).

The ability of a polymer membrane to separate a mixture of two gases is based on the different solubility and diffusivity that each gas shows in comparison to the polymeric matrix and is generally defined as *selectivity*. The selectivity of a membrane for a gas A over a gas B, α_A,B_, is the ratio of the pure gas permeabilities, Equation (4) [35]:

(4) $$\alpha_{A,B}=\frac{P_{A}}{P_{B}}=\frac{D_{A}}{D_{B}}\frac{S_{A}}{S_{B}}.$$

For a binary gas mixture, selectivity can also be defined in terms of the upstream (y) and downstream (x) mole fractions of gas phases, Equation (5) [35]:

(5) αA,B=yAyBxAxB.

From an experimental point of view, the permeability of a gas through a membrane is obtained by measuring the one-dimensional gas flow across the thickness of the membrane under a pressure difference ΔP, between the upstream (high pressure side) and the downstream (low pressure side) of the membrane faces.

The composition of permeate streams was analysed in real time by gas chromatography. The molar composition of the gas mixture, contained in V_2_, was analyzed by an Agilent 6890N gas chromatograph (GC) (Agilent Technologies, Santa Clara, CA, USA) equipped with a flame ionization detector (FID) and a thermal conductivity detector (TCD), using a GS-Alumina column (50 m × 0.55 mm ID). The total acquisition time of the gas chromatographic method for the analysis of the permeate stream was 15 min, using nitrogen as both carrier and balanced gas. From GC analysis results, the membrane selectivity, αH2CH4, is determined. The same experimental setup, when used with pure gas streams, allows to determine transport properties of the membrane in terms of permeability and diffusivity coefficient D by the “time lag” method [34,36].

Each measure was repeated 5 times, and average results are reported; the standard deviation for the mean values is very low, ranging between ±0.1 and 0.3%, ensuring the repeatability of the measurements. The developed materials resulted stable under the operating conditions, after numerous cycles of measurement.

## 3. Results and Discussion

### 3.1. Graphite Substrate Characterization

**Figure 3:**
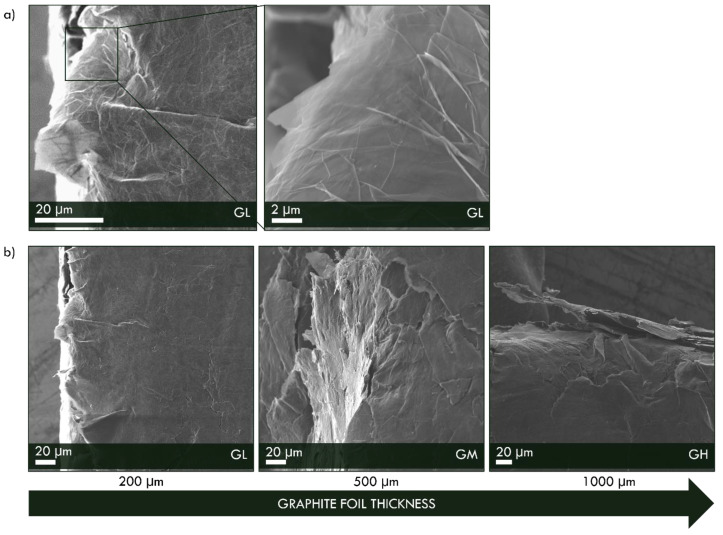
SEM images of (**a**) GL sample at low and high magnificence, (**b**) GL, GM and GH samples.

In Figure 3, the SEM micrographs of the graphite foil used as the membrane substrate are reported. As it clearly appeared, thin and small carbon sheets are pressed together in a compact structure. Despite the different substrate thickness, 1000 μm, 500 μm and 200 μm, the morphology of all graphite foils is similar, as shown in Figure 3.

The mechanical characterization of the graphite support was carried out by the Small Punch Test (SPT) [37,38] as described in the Experimental section. The small punch test makes it possible to evaluate the mechanical behaviour of the separation membranes under stress conditions, similar to the working conditions and certainly more realistic than the classic tensile test.

**Figure 4:**
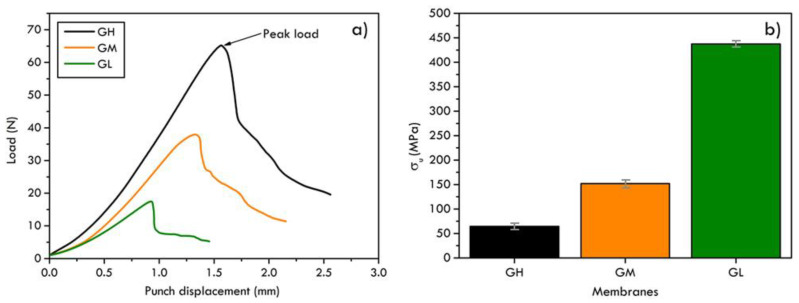
Force–displacement curves (**a**) and estimated values of the tensile strength (**b**) of the three graphite foils with different thicknesses.

In Figure 4a, the force–displacement curves of the graphite foils with different thickness are reported. The comparison shows a similar trend for all the graphite sheets that is characterized by an initial elastic deformation phase and a peak load, indicating the first fracture formation, and a following cracks propagation region at lower loads. It can be concluded that the graphite sheets show a brittle rupture with no evidence of plastic deformation.

The results of the SPT can be used to estimate “conventional” mechanical parameters of materials [37,39]; in particular, the peak load can be related to the ultimate tensile strength, *σ_u_*, (MPa) with the relationship as in Equation (6),

(6) $$\sigma_{u}=\alpha \frac{P_{u}}{t^{2}},$$

where *P_u_* is the peak load (N), *t* the membrane thickness (mm) and *α* a characteristic coefficient of the material.

In Figure 4b, the estimated values of the tensile strength for the three different thicknesses of graphite foils are compared. It is evident that while the membrane thickness decreases, the ultimate strength increases. The observed worsening of the mechanical resistance with the thickness is due to a greater number of structural defects introduced during the overlapping of several layers of graphite. The greater the number of graphite layers, the lower the mechanical resistance of the sheet.

**Figure 5:**
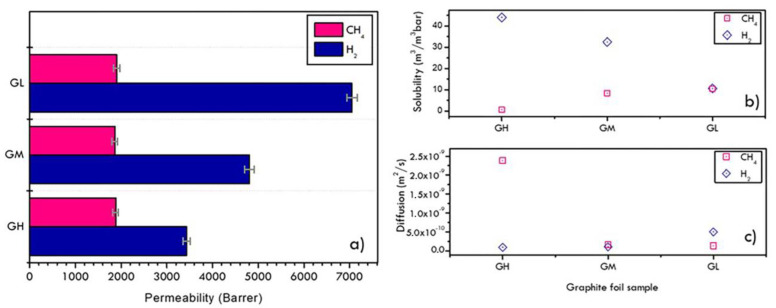
Permeability (**a**), solubility (**b**) and diffusion (**c**) coefficients of pure hydrogen and methane gases over graphite foils at different thickness.

Preliminary results of hydrogen and methane permeabilities suggested the suitability of those membranes for the proposed application. The single-gas permeability of hydrogen and methane was measured at room temperature, and the relative values are shown in Figure 5a. Hydrogen permeabilities in graphite foils showed a sharp increase by decreasing the foil thickness, while the CH_4_ permeability was almost unvaried in all cases, likely linked to different kinetical behaviours. Indeed, the evident lower value of permeability for CH_4_ with respect to H_2_ could be explained by the higher relative adsorption of hydrogen, compared to methane, which has been measured in porous carbons [40,41,42]. The mechanism of gas permeation in graphite sheets seems to be dominated by interlayer spacing rather than through inter-grain defects. Channels formed by interlayer defects have a longer length than inter-grain defects that instead generally act on size-based sieving [43,44]. Consequently, longer channels provide many adsorption sites, thus allowing significant differential absorption of hydrogen with respect to methane. Accordingly, as evidenced in Figure 5c, H_2_ diffusion coefficient increases by decreasing the graphite foil thickness and the solubility parameter follows instead an opposite trend. In particular, the solubility is proportional to the adsorption active sites, as in the case of GH sample, whose better solubility of H_2_ was attributed to the higher number of “adsorption” centres due to a larger number of interlayer defects [40]. On the contrary, in the case of CH_4_, the variation of solubility results less marked with the thickness decrease, while the diffusion coefficient diminishes.

Finally, the graphite foil with the smallest thickness, the GL sample, was chosen as the preferred support for the composite membranes, on account of both its mechanical resistance and better permeation performances compared to the other two sheets.

### 3.2. Polymers/Graphite Membranes

**Figure 6:**
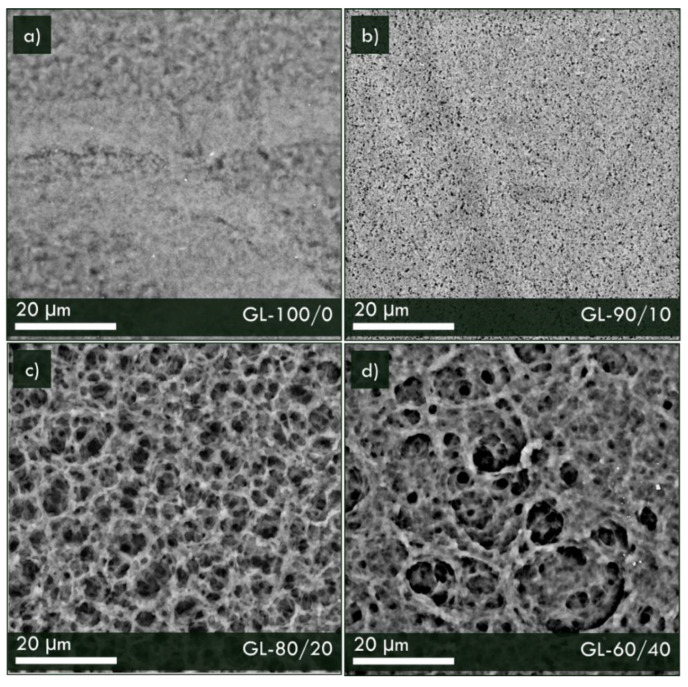
SEM images of top surfaces of composite membranes: (**a**) GL-100/0, (**b**) GL-90/10 (**c**) GL-80/20 and (**d**) GL-60/40 samples.

SEM micrographs of thin solid polymeric membranes, supported over the 200 μm thick graphite foil, are shown in Figure 6. In these pictures, SEM images of the external surfaces of composite membranes are shown. A very different morphology of polymer-based membranes, clearly influenced by the content of the additive, is evidenced upon increasing the Nafion^TM^ content in PVDF-HFP polymeric solution. In particular, sample GL-100/0, a pure PVDF-HFP membrane, is characterized by a spherulitic structure and a smooth surface, suggesting the formation of a compact layer able to completely cover the graphite substrate [27]. The addition of Nafion^TM^ into sample GL-90/10 displayed a homogeneous surface, but smaller spherulite sizes and well-distributed holes were evidenced in this case. Upon increasing Nafion^TM^ concentration, in sample GL-80/20, a superficial porous-like structure was instead revealed and resulted further modified in sample GL-60/40 when the highest Nafion^TM^ concentration was used.

**Figure 7:**
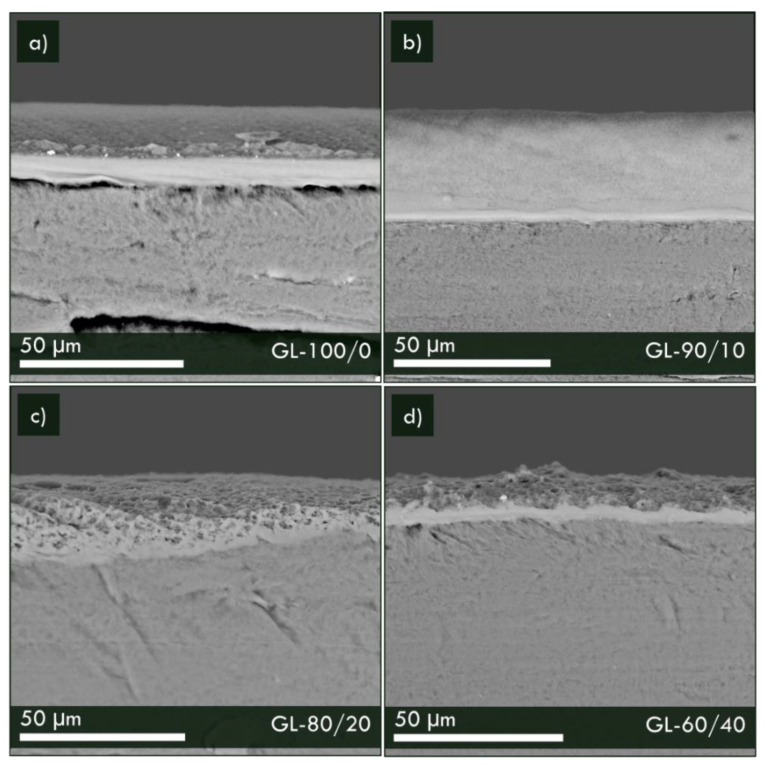
Detailed SEM micrographs of composite membranes in the cross-sectional direction: (**a**) GL-100/0, (**b**) GL-90/10 (**c**) GL-80/20 and (**d**) GL-60/40 samples.

Despite the same tape casting and evaporation procedures, an increased superficial porosity was evidenced and mainly ascribed to the different time of evaporation of mixed solvents due to the different composition of the solution. In addition, variable membrane thickness and cross-section structures were obtained, as shown in Figure 7. The analysis of the interface between the graphite support and the polymeric membranes highlighted their good adhesion, showing a compact interfacial layer for samples GL-100/0, GL-90/10 and GL-60/40 and a peculiar almost single platelet adhesion in the case of sample GL-80/20, suggesting, in this case, a better interaction between the two different layers. Beyond the compact interfacial layer, all the samples showed an increased porosity in the cross-sectional direction as GL-100/0 < GL-90/10 < GL-80/20 < GL-60/40. Superficial porosities and thicknesses of the compact layer of membranes are reported in Table 2.

The thickness of the polymeric membrane plays an important role in gas separation because the diffusion resistance of gases increases, increasing the thickness with the negative consequence of a low permeation rate. Thin polymeric membranes, however, are characterized by low mechanical resistance, which increases the risk of damages and ruptures of the membrane during use. For such a reason, in this work, we proposed a composite matrix membrane where the polymeric layer is supported by a graphite foil so that the thickness of the polymeric membrane was maintained quite low in order to maintain acceptable gas flow, differently from what is generally reported in similar studies, where higher membrane thickness, at least of 100 μm, is reported [27].

**Figure 8:**
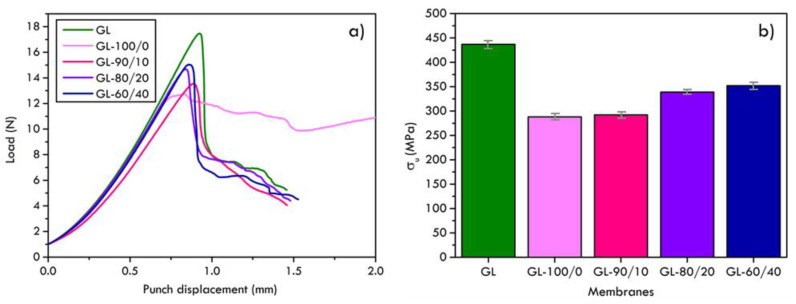
Force–displacement curves and (**a**) estimated values of the tensile strength (**b**) of pure graphite foils and tested samples.

The mechanical behaviour of the composite membranes, measured by SPT, demonstrated that the polymeric layer deposited on the graphite foil GL caused a worsening of the mechanical response of the original graphite sheet, as shown by the peak loads and the estimated tensile strength plots (Figure 8a). Sample GL-100/0, which is obtained depositing a layer of pure PVDF-HFP, showed a region of plastic deformation after the peak load characterized by a crazing phenomenon, formation of micro voids and fibrillae, which is typical in the fracture of thermoplastic polymers. The addition of Nafion^TM^ percentages in the PVDF-HFP matrix (samples GL-90/10, GL-80/20 and GL-60/40) caused an enhancement of the composite membrane strength compared to sample GL-100/0 which increased with the Nafion^TM^ concentration (Figure 8b). The reason for the worsening of the mechanical strength of graphite when coated with a polymeric layer should be attributed to the residual interface stresses arising during the polymeric layer drying after deposition on the graphite foil. The surface between the supporting graphite and the polymeric membrane is, indeed, a region of concentration of stresses that arise because the solvent evaporation is faster at the polymer/air interface compared to the graphite/polymer interface so that the polymeric matrix vitrification (the polymer transition from the solution to the glassy phase) occurs at different times between the surface exposed to the air and the layer in contact with the graphite. Although this interface interaction is the cause of the observed embrittlement, it is also a proof of the good adhesion that develops between the polymeric layer and the supporting graphite during the membrane casting.

**Figure 9:**
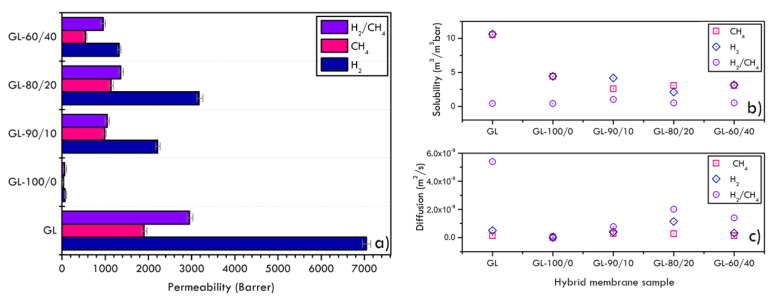
Permeability (**a**), solubility (**b**) and diffusion (**c**) coefficients of pure and mixed gases over investigated membranes.

The gas separation property of polymeric membranes is based on the solution-diffusion mechanism. Polymeric membranes do not have a “continuous” porosity like micro and nano porous materials, i.e., zeolites and MOF [45], so the separation mechanism does not generally occur due to a sieving effect based on the different molecular size of gases, but mainly relies on the diffusion characteristics of gases into the polymeric matrix. A gas molecule can diffuse through an amorphous polymer due to transient gasps, pockets of free volume that are created temporally in the bulk by the thermally agitated motion of chain segments of the polymeric matrix. This free, mobile space allows the gas to dissolve and diffuse from the upstream to the downstream face of the membrane under a pressure gradient between the two sides. Hydrogen, methane and mixed-gas permeability of polymers/graphite samples are reported in Figure 9, together with the plots of solubility and diffusion coefficients of composite membranes, and relative values reported in Table 3. In accordance with morphological results, GL-100/0 membrane resulted the one with the lowest permeability in all the tested conditions, confirming the dense and well-packed polymeric layer highlighted in the SEM micrograph, as also reported by other authors using the same polymer [27]. In order to modify and enhance the permeability, Nafion^TM^ polymer was added, owing to the increase in the amount of the fractional free volume, based on the free volume theory that correlates the gas diffusion to the available free volume within a polymer matrix [27,46]. Indeed, increasing polymer chain mobility induces the reorganization of local segmental chains, finally promoting the gas diffusivity in the polymer [47,48]. The addition of low concentration of Nafion^TM^ promoted the described mechanism and a slight increase in both hydrogen and methane permeabilities was registered in sample GL-90/10, then reached the maximum values in GL-80/20 and finally decreased in GL-60/40 sample, hindering a threshold value for the additive content (Figure 9). As extensively discussed, permeability is a function of both gas solubility and diffusivity. It should be further highlighted that gas permeability in rubbery polymers, as is the case of PVDF-HFP, characterized by a glass transition temperatures (Tg) below the operating temperature, is lead principally by gas condensability, related to the critical temperature, and characterized by high values of the diffusion coefficients due to the molecular chain mobility and the high free volume; on the contrary, in glassy polymers, with Tg above the operating temperature, due to the kinetic hindrance of chains relaxation, a rigid polymeric lattice is present, so that diffusivity is mainly related to the size difference between the gas molecules and the size sieving ability of the polymer [49,50]. The presence of Nafion^TM^, a glassy polymer in the defined experimental conditions, favoured the H_2_ permeability which in turn resulted always higher than CH_4_ for all the membranes, as also reported by other authors evidencing the same permeability trend, even in different temperature and feed pressure conditions [27,50]. This was also likely correlated with the kinetic diameter of the gases with a smaller H_2_ diameter (0.289 nm) than that of CH_4_ (0.380 nm). However, the kinetic diameters are related not only to the gas molecular size but also to their molecular structure [40].

Accordingly, binary (1:1) H_2_/CH_4_ mixed-gas permeation experiments were conducted to evaluate the potential effectiveness of Nafion^TM^ in separating H_2_/CH_4_ mixtures. Indeed, for gas mixtures, the transport behaviour of one component through the membrane is affected by the presence of the other, resulting in lower permeability values compared to pure gases. Therefore, the membrane performance during mixed-gas measurements must be analysed [50,51] since the evaluation of membrane behaviour under gas mixtures using single gas permeation may lead to misleading results. Overall, mixed-gas H_2_/CH_4_ permeabilities were lower than pure-gas ideal permeabilities, as it has been observed by others in the literature, even with other gas pairs [50]. In particular, competitive sorption effect led to a drop in permeability with respect to H_2_ gas and a slight gain with respect to pure CH_4_ values.

In regard to solubility and diffusion coefficients of composite membranes, by data comparison, it is evident that solubilities did not change significantly, increasing Nafion^TM^ content while gas diffusivity first increased, then achieved a maximum value corresponding to the best additive quantity and finally diminished again. Since permeability is the product of diffusivity and solubility, the constant solubility values indicate that the variation in permeabilities was more influenced by the diffusivity of gases. This behaviour was ascribed to the polymers chain compression and relaxation that, acting on the free volume, narrowed gas transport pathways, reducing larger gas molecules contribution [28].

**Figure 10:**
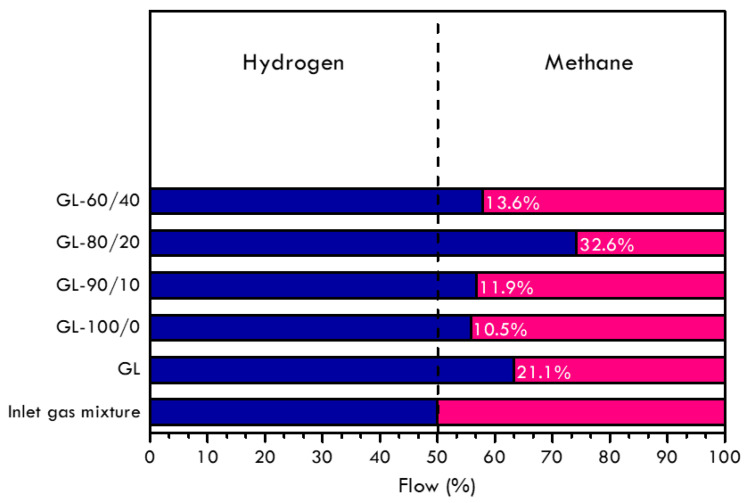
Outlet gas mixtures composition (v%).

Together with permeability values, the H_2_ enrichment of the outlet streams is the other important parameter that was analysed because it is directly correlated to the selectivity of the different membranes prepared. Indeed, despite the lowering of the permeability of the polymers/graphite membranes with respect to the pure graphite foil, the H_2_ enrichment followed a different trend, favouring the selectivity of the hybrid membranes, as shown in Figure 10. In this graph, the bars represent the composition of the outlet gas mixtures compared to the upstream flow, and the enrichment in hydrogen reached by each membrane. The inlet gas mixture was fixed as 1:1 ratio of hydrogen and methane, respectively, and the enrichment of hydrogen in the outlet gas flow was evaluated by gas chromatography. In particular, sample GL-80/20 resulted the most effective in improving the hydrogen separation against methane showing an enrichment of 32.6% of hydrogen in the downstream mixture. The Nafion^TM^ addition of up to 10%, sample GL-90/10, did not demonstrate a significant improvement compared to sample GL-100/0, and already at 40% of Nafion^TM^, the separation capability of the membrane decreased (Figure 10).

These results are in agreement with the permeability values in Figure 9 and the modification of the PVDF-HFP membrane structure by the introduction of a proper quantity of additive. It is interesting to note that the graphite foil GL itself has a positive effect in terms of hydrogen enrichment of the outlet stream due to the cited affinity to graphite of H_2_ compared to CH_4_. The deposition of a polymeric membrane on the GL surface, indeed, determined an improvement only for sample GL-80/20. It can be concluded that in the composite membrane, the polymer layer has been shown to prevail over the properties of graphite in terms of transport properties (Figure 9) and separation effects (Figure 10).

**Figure 11:**
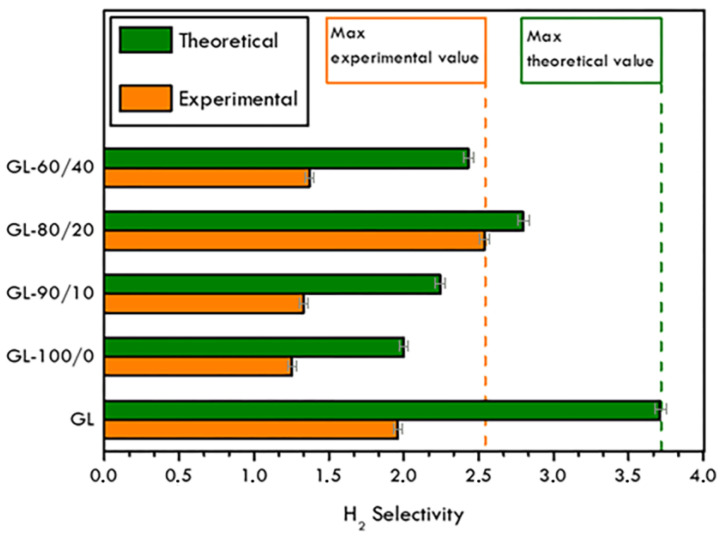
Theoretical and experimental H_2_ selectivity values for analysed samples.

In Figure 11, the separation properties of membranes are reported in terms of the selectivity αH2CH4, upstream and downstream mole fractions of gases, calculated by the concentration data from the chromatography analysis (experimental values) compared to theoretical selectivity α obtained from the ratio of permeabilities of pure hydrogen and methane measured for each membrane.

As expected, due to different experimental permeability values and the selectivity, important deviations between theoretical and mixed-gas values were registered, mainly due to the CH_4_ accumulation in the upstream flow. The theoretical value is always higher than the experimental one, hindering with the fact that mixed-gas permeability is highly influenced by the kinetic of the mixed-gas flow, as previously discussed for permeability. Moreover, experimental data are of the same order of magnitude of the data reported in the literature [52], and is even improved for similar analysed system, unless with the use of room temperature but under different feed pressure condition, which was decisively milder in the present study.

## 4. Conclusions

This work reports new findings on polymeric fluoro-based hybrid membranes supported on graphite foils that were produced, characterized and tested towards gas separation performance. Hybrid membranes were realized using the doctor blade technique and were investigated in depth by complementary characterization analysis. The suitability of the graphite support, especially in term of thickness, was first investigated and assessed. PVDF-HFP polymer, already experimented on in gas separation membranes, proved its ability to interact with both the graphite support and Nafion^TM^, was used as an additive. The peculiar morphology of the obtained materials resulted to be greatly influential not only on the gas separation properties but also the mechanical behaviour of membranes, evaluated reproducing the testing conditions. The permeability of pure gases (H_2_ and CH_4_) as well as their mixtures was studied and the theoretical selectivity values were calculated and compared to the experimental ones, obtained through gas chromatography. Numerous samples were fabricated, optimized and tested, owing to increase in the H_2_ selectivity when hydrogen/methane gas mixtures were considered. Preliminary investigations on those hybrid membranes show their ability to work in very mild conditions, such as near atmospheric pressure and at room temperature, in the selective separation of hydrogen from methane, differently from traditional separation membranes, characterized instead by more critical operating conditions and high costs. In the most performing developed membrane, a H_2_ enrichment equal to 32.6% (v%) was registered. Moreover, the experimental selectivity value α = 2.54 was quite in agreement with the theoretical one.

## Figures and Tables

**Table 1:** Sample composition.

Sample	Substrate	Composition (wt%)			
		PVDF-HFP	Acetone	Nafion^TM^	Water + Lower Aliphatic Alcohols
GH	Graphite 1000 μm	-	-	-	-
GM	Graphite 500 μm	-	-	-	-
GL	Graphite 200 μm	-	-	-	-
GL-100/0	Graphite 200 μm	5.0	95.0	-	-
GL-90/10	Graphite 200 μm	4.5	85.5	0.5	9.5
GL-80/20	Graphite 200 μm	4.0	76.0	1.0	19.0
GL-60/40	Graphite 200 μm	3.0	57.0	2.0	38.0

**Table 2:** Polymeric membranes characteristic: superficial porosity and thickness.

Membrane	Superficial Porosity (%)	Thickness (mm)
GL-100/0	8.34	9.16 ± 0.2
GL-90/10	14.13	8.43 ± 0.2
GL-80/20	49.04	3.12 ± 0.2
GL-60/40	49.02	3.30 ± 0.2

**Table 3:** Membrane characteristics: diffusion and solubility coefficients.

Samples	Diffusion (m^2^/s)			Solubility (m^3^/m^3^bar)		
	H_2_/CH_4_	H_2_	CH_4_	H_2_/CH_4_	H_2_	CH_4_
GL	5.39 × 10^−9^	5.04 × 10^−10^	1.37 × 10^−10^	0.42	10.64	10.52
GL-100/0	1.00 × 10^−10^	1.17 × 10^−11^	5.84 × 10^−12^	0.43	4.42	4.42
GL-90/10	7.80 × 10^−10^	4.03 × 10^−10^	2.90 × 10^−10^	1.02	4.18	2.59
GL-80/20	2.02 × 10^−9^	1.15 × 10^−9^	2.81 × 10^−10^	0.51	2.10	3.08
GL-60/40	1.40 × 10^−9^	3.19 × 10^−10^	1.34 × 10^−10^	0.52	3.16	3.09
